# The relation between serum D-dimer, ferritin and vitamin D levels, and dysgeusia symptoms, in patients with coronavirus disease 2019

**DOI:** 10.1017/S0022215120002765

**Published:** 2021-01-07

**Authors:** E Elibol, H Baran

**Affiliations:** 1 Department of Ear, Nose and Throat Diseases, Yenimahalle Education and Research Hospital, Ankara Yıldırım Beyazıt University, Ankara; 2 Department of Ear, Nose and Throat Diseases, Istanbul Kartal Dr Lutfi Kirdar Training and Research Hospital, University of Health Sciences, Istanbul, Turkey

**Keywords:** Anosmia, Coronavirus, Pandemics, Smell Disorders

## Abstract

**Objective:**

This study aimed to evaluate the association between serum D-dimer, ferritin and vitamin D levels, and dysgeusia symptoms, in patients with coronavirus disease 2019.

**Methods:**

The present study was conducted with the medical records of 300 patients positive for coronavirus disease 2019, hospitalised between 28 March and 15 August 2020. The patients were divided into two groups regarding the presence or absence of dysgeusia symptoms.

**Results:**

Fever and sore throat rates, and the mean D-dimer level, were considerably higher in the dysgeusia group than in the non-dysgeusia group (32.1 *vs* 21.6 per cent, *p* = 0.04; 43.6 *vs* 20.7 per cent, *p* < 0.001; and 0.54 ± 0.32 *vs* 0.49 ± 0.51 mg/l FEU, *p* = 0.008, respectively). The mean age was significantly lower in the dysgeusia group than in the non-dysgeusia group (42.83 ± 12.31 *vs* 50.51 ± 13.67 years, *p* < 0.001).

**Conclusion:**

Younger age, fever and shortness of breath could be observed in patients with dysgeusia symptoms. In addition, the D-dimer level was significantly higher in the dysgeusia group.

## Introduction

The outbreak of coronavirus disease 2019 (Covid-19), which is caused by the highly contagious severe acute respiratory syndrome coronavirus 2 (SARS-CoV-2), was announced a pandemic in March 2020 by the World Health Organization.^1^ The disease mainly affects the respiratory system and spreads via aerosols released during sneezing and coughing.^2^ The main symptoms of Covid-19 are fever, cough, runny nose, nasal congestion, shortness of breath, headache and myalgia.^2,3^ The disease can be diagnosed on the basis of clinical symptoms, polymerase chain reaction positivity and the presence of ground-glass opacities on computed tomography (CT) scans.^4^

Recent studies have focused on the role of serum inflammatory markers that predict Covid-19, such as lymphocyte counts and C-reactive protein (CRP), homocysteine and D-dimer levels.^5,6^ The levels of ferritin, a crucial immune response mediator, increase in severe Covid-19 cases.^7^ Increased ferritin levels could cause a cytokine storm by exerting direct immunosuppressive and pro-inflammatory effects.^7^ Vitamin D, which affects the nuclear vitamin D receptor, enhances innate cellular immunity by inducing antimicrobial peptides.^8^ Vitamin D is thought to reduce the risk of viral infections through several mechanisms, and decreased vitamin D levels have been observed in patients with viral pneumonia.^8^ D-dimer is a fibrin degradation product used to exclude the diagnosis of thrombosis. Increased D-dimer levels have been observed in severe Covid-19 cases accompanied by microangiopathy and a hypercoagulable state.^9^

In addition to the typical Covid-19 symptoms, recent otolaryngology literature has focused on the association between anosmia and dysgeusia symptoms and the development of minimal-to-mild Covid-19.^10^ The assessment of these symptoms does not require any intervention and is easy to perform. Yan *et al*. reported that patients with taste disorders have a 10-fold higher risk of developing Covid-19.^11^ However, there is a lack of evidence about the association between the levels of serum markers predicting disease severity and dysgeusia symptoms.^11^

The present study aimed to evaluate the association between serum D-dimer, ferritin and vitamin D levels, and dysgeusia symptoms, in patients with Covid-19.

## Materials and methods

The clinical data of 300 patients confirmed to have Covid-19 via real-time reverse transcription polymerase chain reaction, who were hospitalised in a tertiary referral hospital between 28 March and 15 August 2020, were analysed. Ethical approval was obtained from the National Ministry of Health (Covid-19 study registration system) (approval number: 2020-05-04T08-34-29).

The Turkish national guidelines recommend that patients with persistent fever and a history of contact with a Covid-19 positive individual undergo a polymerase chain reaction test.^12^ The present study included patients aged over 18 years who were confirmed to have Covid-19 based on real-time reverse transcription polymerase chain reaction assessment of oropharyngeal and nasopharyngeal samples. Patients who had vomiting, diarrhoea, weakness, systemic diseases and lower oxygen saturation were hospitalised. Patients with previous olfactory or gustatory dysfunction, without polymerase chain reaction confirmed Covid-19, with a history of thromboembolism, with vitamin D deficiency, receiving vitamin D or ferritin treatment, or with a history of bowel resection, were excluded from the study.

The patients’ clinical data, including age, gender, systemic co-morbidities (e.g. diabetes mellitus, hypertension, chronic obstructive pulmonary disease and asthma) and intensive care unit admission, were recorded. Moreover, patients’ symptoms, such as cough, shortness of breath, rhinorrhoea, sore throat, loss of smell, dysgeusia, fever and vomiting, were documented. The patients were divided into two groups: without dysgeusia and with dysgeusia.

All patients underwent a CT scan (BrightSpeed CT scanner; GE Healthcare Systems, Milwaukee, Wisconsin, USA) according to national guideline recommendations for management of the disease. The presence of ground-glass opacities on CT scans was recorded.^4^

Supportive care was provided and hydroxychloroquine was administered (two doses of 200 mg twice daily, followed by 100 mg twice daily for 5 days). Favipiravir (1600 mg by mouth on admission, followed by 600 mg by mouth for 5 days) and enoxaparin (0.4 IU daily, by subcutaneous injection) were also administered when needed.^12^

### Statistical analysis

Data were analysed using SPSS statistical software, version 20.0 (SPSS, Chicago, Illinois, USA). All continuous data are presented as means and standard deviations, while categorical data are presented as numbers and percentages. The Shapiro–Wilk test was used to analyse the distribution of continuous variables. The student's *t*-test was used to analyse parametric variables, while the Mann–Whitney U test was used to analyse non-parametric variables. A chi-square test was used to compare categorical variables.

Multivariate regression analysis was performed to analyse relationships between age, white blood cell (WBC) count, CRP level, D-dimer level, fibrinogen level and vitamin D level, and dysgeusia symptoms. A primary regression model was generated using a stepwise procedure and included all potential interaction variables. This model was generated using independent variables achieving a *p*-value of 0.10 during bivariate analysis. Then, the best-fit model was generated without interaction variables. The presence of dysgeusia symptoms was considered to be the dependent variable.

For all calculations, a *p*-value of less than 0.05 was considered statistically significant.

## Results

The mean age of the study population was 48.52 ± 13.73 years. Of the patients, 54.3 per cent were male. The most common co-morbidities were hypertension (5.3 per cent) and pulmonary disease (5 per cent). The most common presenting symptoms were cough (34.3 per cent), sore throat (26.7 per cent), fever (24.3 per cent), vomiting (23.7 per cent) and shortness of breath (20.3 per cent). In total, 181 patients (60.3 per cent) had Covid-19 findings on CT scans. Regarding the accompanying symptoms, 104 patients (34.7 per cent) had anosmia and 78 (26 per cent) had dysgeusia (Table 1).

**Table 1.** Clinical characteristics and presenting symptoms of study population

Parameter	Value
Age (mean ± SD; years)	48.52 ± 13.73
Gender (*n* (%))	
– Female	137 (45.7)
– Male	163 (54.3)
Systemic disease (*n* (%))	
– None	237 (79)
– Hypertension	16 (5.3)
– Diabetes mellitus	8 (2.7)
– Hypertension + diabetes mellitus	4 (1.3)
– Pulmonary disease	15 (5)
– Other	20 (6.7)
Fever (*n* (%))	
– No	227 (75.7)
– Yes	73 (24.3)
Cough (*n* (%))	
– No	197 (65.7)
– Yes	103 (34.3)
Sore throat (*n* (%))	
– No	220 (73.3)
– Yes	80 (26.7)
Rhinorrhoea (*n* (%))	
– No	282 (94)
– Yes	18 (6)
Shortness of breath (*n* (%))	
– No	239 (79.7)
– Yes	61 (20.3)
Vomiting (*n* (%))	
– No	229 (76.3)
– Yes	71 (23.7)
Anosmia (*n* (%))	
– No	196 (65.3)
– Yes	104 (34.7)
Dysgeusia (*n* (%))	
– No	222 (74)
– Yes	78 (26)
CT positivity (*n* (%))	
– No	119 (39.7)
– Yes	181 (60.3)

SD = standard deviation; CT = computed tomography

The mean patient age was significantly higher in the group without dysgeusia than in the group with dysgeusia (50.51 ± 13.67 years *vs* 42.83 ± 12.31 years, *p* < 0.001). The most commonly observed co-morbidity was hypertension, in both the groups; the incidence rates of systemic co-morbidities did not differ significantly between the groups. Regarding the patients’ symptoms, the incidence of fever was significantly higher in the dysgeusia group than in the non-dysgeusia group (32.1 per cent *vs* 21.6 per cent, *p* = 0.04). Moreover, the incidence of sore throat was significantly higher in the dysgeusia group than in the non-dysgeusia group (43.6 per cent *vs* 20.7 per cent, *p* < 0.001). On the other hand, no significant differences were observed in the incidence rates for cough, rhinorrhoea, shortness of breath, vomiting or anosmia between the two groups.

The number of patients with CT positivity in the group without dysgeusia (141 (63.5 per cent)) was significantly higher than that in the group with dysgeusia (40 (51.3 per cent)) (*p* = 0.03). No significant differences were observed in terms of the intensive care unit admission rate, oxygen saturation level or duration of hospitalisation between the two groups (Table 2).

**Table 2.** Comparison of clinical characteristics and presenting symptoms of study groups

Parameter	Without dysgeusia	With dysgeusia	*P-*value
Age (mean ± SD; years)	50.51 ± 13.67	42.83 ± 12.31	<0.001*
Gender (*n* (%))			0.08
– Female	107 (48.2)	30 (38.5)	
– Male	115 (51.8)	48 (61.5)	
Systemic disease (*n* (%))			0.334
– None	176 (79.3)	61 (78.2)	
– Hypertension	12 (5.4)	4 (5.1)	
– Diabetes mellitus	7 (3.2)	1 (1.3)	
– Hypertension + diabetes mellitus	4 (1.8)	0 (0)	
– Pulmonary disease	8 (3.6)	7 (9)	
– Other	15 (6.8)	5 (6.4)	
Fever (*n* (%))			0.04*
– No	174 (78.4)	53 (67.9)	
– Yes	48 (21.6)	25 (32.1)	
Cough (*n* (%))			0.472
– No	145 (65.3)	52 (66.7)	
– Yes	77 (34.7)	26 (33.3)	
Sore throat (*n* (%))			<0.001*
– No	176 (79.3)	44 (56.4)	
– Yes	46 (20.7)	34 (43.6)	
Rhinorrhoea (*n* (%))			0.524
– No	209 (94.1)	73 (93.6)	
– Yes	13 (5.9)	5 (6.4)	
Shortness of breath (*n* (%))			0.333
– No	175 (78.8)	64 (82.1)	
– Yes	47 (21.2)	14 (17.9)	
Vomiting (*n* (%))			0.109
– No	165 (74.3)	64 (82.1)	
– Yes	57 (25.7)	14 (17.9)	
Anosmia (*n* (%))			0.169
– No	149 (67.1)	47 (60.3)	
– Yes	73 (32.9)	31 (39.7)	
CT positivity (*n* (%))			0.03*
– No	81 (36.5)	38 (48.7)	
– Yes	141 (63.5)	40 (51.3)	
ICU admission (*n* (%))			0.618
– No	213 (95.9)	75 (96.2)	
– Yes	9 (4.1)	3 (3.8)	
Oxygen saturation (mean ± SD; %)	95 ± 2.01	94.6 ± 2.13	0.522
Hospital stay (mean ± SD; days)	6.4 ± 3.2	6.5 ± 3.2	0.238

*Indicates statistical significance (*p* < 0.05). SD = standard deviation; CT = computed tomography; ICU = intensive care unit

Regarding the WBC count, CRP level, ferritin level, vitamin D level and the presence of dysgeusia symptoms, no significant differences were observed between the two groups. However, the D-dimer levels were significantly higher in the dysgeusia group than in the non-dysgeusia group (0.54 ± 0.32 mg/l fibrinogen-equivalent units (FEU) *vs* 0.49 ± 0.51 mg/l FEU, *p* = 0.008; Table 3).

**Table 3.** Comparison of laboratory findings for study population

Parameter	Without dysgeusia	With dysgeusia	*P*-value
WBC (10^3^/μl)	6.37 ± 4.22	6.53 ± 2.56	0.213
CRP (mg/dl)	18.47 ± 26.03	15.86 ± 17.52	0.343
D-dimer (mg/l FEU)	0.49 ± 0.51	0.54 ± 0.32	0.008*
Ferritin (ng/ml)	151.98 ± 197.18	131.84 ± 144.11	0.583
Vitamin D (ng/ml)	53.68 ± 28.25	51.93 ± 27.43	0.458

Data represent mean ± standard deviation values, unless indicated otherwise. *Indicates statistical significance (*p* < 0.05). WBC = white blood cells; CRP = C-reactive protein

Binary logistic regression analysis revealed that patients’ age (odds ratio = 0.95, 95 per cent confidence interval (CI) = 0.93–0.97) and D-dimer levels (odds ratio = 0.99, 95 per cent CI = 1.06–4.57) were significantly related to the presence of dysgeusia symptoms (Table 4).

**Table 4.** Binary logistic regression analysis of age and serum markers, and dysgeusia symptoms

Parameter	B (SE)	*P*-value	Odds ratio	95% CI	
				Lower	Upper
Age (years)	−0.048 (0.011)	<0.001*	0.95	0.93	0.97
CRP (mg/dl)	−0.006 (0.008)	0.47	0.99	0.97	1.01
WBC (10^3^/μl)	−0.001 (0.38)	0.98	0.99	0.92	1.07
D-dimer (mg/l FEU)	0.793 (0.372)	0.03*	0.99	1.06	4.57
Ferritin (ng/ml)	−0.001 (0.001)	0.46	0.99	0.99	1.001
Vitamin D (ng/ml)	−0.002 (0.005)	0.73	0.58	0.98	1.008

SE = standard error; CI = confidence interval; CRP = C-reactive protein; WBC = white blood cells

## Discussion

In the present study, the patients with dysgeusia were significantly younger than those without dysgeusia. In addition, the incidence rates for fever and shortness of breath, and the D-dimer levels, were considerably higher in the dysgeusia group. However, CT positivity was significantly higher in the group without dysgeusia.

Previous studies have revealed that olfactory and gustatory dysfunctions could be associated with the development of Covid-19.^13,14^ According to these studies, the infectious process of the virus begins by colonising the olfactory and gustatory epithelia, and then turns into an inflammatory process.^10,13^ Avcı *et al*. reported incidence rates for anosmia and dysgeusia symptoms in patients with Covid-19 of 44.2 per cent and 43.9 per cent, respectively.^15^ These authors conducted a study with one of the largest sample sizes, and reported that anosmia was associated with out-patient follow up rather than in-patient management.^15^ In the present study, 34.7 per cent of the patients presented with loss of smell and 26 per cent presented with dysgeusia symptoms. The patients in the current study were divided on the basis of the presence of dysgeusia symptoms rather than anosmia symptoms, because the latter could also be affected by other virus-related infections.

The early diagnosis of Covid-19 and risk stratification of the disease may reduce morbidity and mortality rates.^16^ However, because of non-specific disease symptoms, and false-negative and delayed polymerase chain reaction results, additional markers are needed for the diagnosis and surveillance of the disease.^10^

A study involving 20 patients with Covid-19 evaluated ferritin, a serum inflammatory marker, and reported increased serum ferritin levels in patients with severe and very severe Covid-19, with the levels being significantly higher in the very severe group.^17^ Another study involving 69 patients with severe Covid-19 reported significantly higher ferritin levels in patients with severe disease than in those with non-severe disease.^18^ In addition, the serum marker vitamin D has been evaluated in patients with Covid-19. However, no significant decrease in vitamin D levels has been observed in comparative studies.^8^ In the present study, no significant difference was observed in ferritin or vitamin D levels between the patient groups. We postulate that these results are related to the small and heterogeneous number of severe Covid-19 cases.

Regarding the hypercoagulable state observed in patients with Covid-19, recent studies have evaluated the serum levels of D-dimer, a fibrin degradation product.^9^ Increased D-dimer levels have been reported in 3.75–68.0 per cent of patients with Covid-19; D-dimer levels above 1 μg/ml are accepted as a risk factor for increased mortality in adults.^11^ Moreover, Yao *et al*. reported an association between D-dimer levels and disease severity.^11^ In the present study, D-dimer levels were significantly increased in both groups; however, the levels were higher in patients with dysgeusia symptoms. The increased D-dimer levels in patients with dysgeusia symptoms could be associated with microvascular thrombosis that may be observed in highly vascularised taste buds located on the tongue.

• Severe acute respiratory syndrome coronavirus-2 disease is a highly contagious virus
• Recent otolaryngology literature has focused on the association between anosmia and dysgeusia symptoms
• Younger age, fever and shortness of breath were observed in patients with dysgeusia symptoms
• The D-dimer level was significantly higher in the dysgeusia group

Recent studies have evaluated various serum markers, such as lymphocyte counts, CRP and procalcitonin levels, and erythrocyte sedimentation rate (ESR), to obtain a reliable and easily accessible diagnostic tool, as an alternative to polymerase chain reaction and chest CT scans, and to predict disease severity.^5,6,16^ Recently, Yasui *et al*. reported that decreased levels of zinc, an activator of immune cells that regulate cell-mediated immunity, could be related to Covid-19 severity.^19^ Moreover, Tan *et al*. reported elevated CRP levels and ESR at a very early stage of Covid-19.^6^ They suggested that elevated CRP levels and ESR could predict the occurrence of severe disease before CT findings.^6,20^ However, in the present study, no significant difference was observed in the serum inflammatory markers between the patient groups. This could be due to the heterogeneity of the study population and the different reactions of the host immune response.

Cough (76 per cent) and dyspnoea (55 per cent) are commonly reported symptoms of Covid-19.^14,21,22^ This could be associated with severe pulmonary alveoli damage, which can be detected on CT. In the present study, the most common symptoms were cough (34.3 per cent), sore throat (26.7 per cent) and fever (24.3 per cent). However, no significant differences were observed between the patient groups, except for the incidence of fever and sore throat. Sixty per cent of the patients in our study had CT positivity. The CT positivity was higher in the group without dysgeusia than in the group with dysgeusia. We speculate that the lower CT positivity and the presence of typical symptoms are associated with mild Covid-19 in our study population.

The strength of the present study is that it included a large group of patients diagnosed with Covid-19. However, the study has several limitations. First, oropharyngeal and nasopharyngeal samples were assessed using real-time reverse transcription polymerase chain reaction; false-positive results are possible. Second, our study included heterogeneous Covid-19 cases. Long-term follow-up data are lacking.

In conclusion, the present study demonstrated that patients with dysgeusia symptoms might present with fever, shortness of breath and elevated serum D-dimer levels. The increased hypercoagulable state may cause loss of taste in patients with Covid-19. Further studies should be conducted to evaluate D-dimer levels and dysgeusia symptoms in patients with minimal, mild and severe Covid-19 using objective assessment methods.
